# Mitochondrial DNA deletions in muscle satellite cells: implications for therapies

**DOI:** 10.1093/hmg/ddt327

**Published:** 2013-07-11

**Authors:** Sally Spendiff, Mojgan Reza, Julie L. Murphy, Grainne Gorman, Emma L. Blakely, Robert W. Taylor, Rita Horvath, Georgia Campbell, Jane Newman, Hanns Lochmüller, Doug M. Turnbull

**Affiliations:** 1 Wellcome Trust Centre for Mitochondrial Research, The Medical School, Newcastle University, Newcastle upon Tyne NE2 4HH, UK; 2 Department of Kinesiology and Physical Education, McGill University, Montreal H2W 1S4, Canada; 3 Institute of Genetic Medicine, Newcastle University, Newcastle upon Tyne NE1 3BZ, UK

## Abstract

Progressive myopathy is a major clinical feature of patients with mitochondrial DNA (mtDNA) disease. There is limited treatment available for these patients although exercise and other approaches to activate muscle stem cells (satellite cells) have been proposed. The majority of mtDNA defects are heteroplasmic (a mixture of mutated and wild-type mtDNA present within the muscle) with high levels of mutated mtDNA and low levels of wild-type mtDNA associated with more severe disease. The culture of satellite cell-derived myoblasts often reveals no evidence of the original mtDNA mutation although it is not known if this is lost by selection or simply not present in these cells. We have explored if the mtDNA mutation is present in the satellite cells in one of the commonest genotypes associated with mitochondrial myopathies (patients with single, large-scale mtDNA deletions). Analysis of satellite cells from eight patients showed that the level of mtDNA mutation in the satellite cells is the same as in the mature muscle but is most often subsequently lost during culture. We show that there are two periods of selection against the mutated form, one early on possibly during satellite cell activation and the other during the rapid replication phase of myoblast culture. Our data suggest that the mutations are also lost during rapid replication *in vivo*, implying that strategies to activate satellite cells remain a viable treatment for mitochondrial myopathies in specific patient groups.

## INTRODUCTION

Mitochondrial myopathies are an important group of muscle conditions caused by defects in mitochondrial oxidative phosphorylation. Patients often present with muscle weakness, although fatigue and exercise intolerance are frequently a problem. In some patients, the predominantly proximal myopathy is accompanied by severe involvement of the extraocular muscles leading to chronic progressive external ophthalmoplegia (CPEO) (1), while in others it is part of a multi-system disorder. The myopathic symptoms are progressive and disabling in many patients. Unfortunately, there is little in the way of specific treatment.

Primary mutations of mitochondrial DNA (mtDNA) are a common cause of mitochondrial myopathy and may be either point mutations or single, large-scale mtDNA deletions. MtDNA is present in tissues in multiple copies and the mtDNA mutations causing mitochondrial myopathies are frequently heteroplasmic, with a mixture of mutated and wild-type forms (2–4). The vast majority of mtDNA mutations are functionally recessive and a biochemical deficit is only observed when there are high levels of mutated mtDNA and low levels of wild-type mtDNA (5,6). Heteroplasmic mtDNA defects classically give rise to the mosaic pattern of cytochrome *c* oxidase (COX) normal, intermediate and deficient muscle fibres (7–9).

Heteroplasmic mtDNA mutations may be maternally inherited or occur sporadically. Inherited point mutations often have a widespread tissue distribution and are rarely restricted to a single tissue, although the levels may vary between these tissues. Sporadic mtDNA mutations, either point mutations or single, large-scale mtDNA deletions, are likely to be at much higher levels in post-mitotic tissues such as muscle when compared with replicating tissue (2,10,11). Even when single, large-scale mtDNA deletions are found in replicating tissues such as blood, the mutations tend to be lost rapidly (12,13), implying a selection against cells with a biochemical deficiency on account of high levels of mutation.

The treatment of patients with mitochondrial myopathies remains extremely challenging. Several groups have investigated patients with sporadically occurring mtDNA mutations to determine whether it might be possible to employ a novel approach to therapy by stimulating the growth of muscle stem cells (satellite cells). Previous observations have demonstrated that the disease-causing mtDNA defect is absent in cultured myoblasts of patients with sporadically occurring mtDNA mutations, suggesting that the mtDNA defect is not present, or is very low in satellite cells (14–16). Satellite cells lie on the muscle fibre underneath the basal lamina and share a common dermomyotome embryological origin with developing muscle progenitor cells (17). They are responsible for muscle growth, repair and hypertrophy. In response to activation, they form proliferating myoblasts which differentiate into myotubes and fuse into the muscle fibre to repair it or contribute to hypertrophy (18). Satellite cells can be identified by their expression of transcription factors (e.g. Pax 7) and adhesion molecules [e.g. neural cell adhesion molecule (NCAM) and M-cadherin] (19). There has, however, been considerable debate regarding what constitutes ‘the true muscle satellite cell’, with evidence suggesting that the satellite cell population is heterogeneous based on marker expression (20). In addition, the majority of research involving satellite cells occurs in rodents and the markers have been shown to differ from those in humans (21).

A number of studies have attempted to shift the levels of wild-type and mutated mtDNA in the muscles of affected patients. Two early studies, which involved either injecting myotoxic bupivacaine hydrochloride or trauma induced by re-biopsying to damage muscle, were successful in favourably changing the mtDNA balance and characteristics of regenerating muscle fibres (22,23). While these approaches showed promise, they were not practical and a less severe method of inducing satellite cell activation was required. More recently, resistance exercise training has been evaluated as a method of damaging muscle fibres and inducing satellite cell activation. These experiments have demonstrated marked improvements in mitochondrial activity in muscle as evidenced by a decrease in the proportion of COX-deficient fibres (fibres with low mitochondrial activity) but with inconsistent outcomes on the mtDNA genotype (24,25).

Single, large-scale mtDNA deletions are common causes of mitochondrial myopathies with patients presenting with either a proximal myopathy with associated CPEO, or with a more systemic illness such as Kearns–Sayre syndrome. To establish if activating satellite cells is a viable treatment in these patients, we investigated the differences in deletion load between muscle satellite cells and mature muscle in eight patients. Both satellite cells and muscle have the same embryological origin and, even though experiments *in vitro* and *in vivo* have suggested that myoblasts have low or absent levels of mutation, understanding the mechanism of this remarkable difference compared with the mature muscle might facilitate different therapeutic strategies to help patients with mitochondrial myopathies.

## RESULTS

### Patients and samples

Samples were collected from a total of eight patients (Table 1). For some patients (1, 2, 3, 4, 5 and 7), repeat muscle biopsy samples were available, whereas for others (6 and 8) tissue from only a single biopsy was taken.

**Table 1.** Clinical and molecular genetic characteristics of our patient cohort

Patient	Sex	Age	Age at onset of disease	Symptoms	MtDNA deletion size (bp)	MtDNA deletion breakpoints
1	F	58	37	CPEO, EI, fatigue	3693	nt.9756-nt.13449
2	M	46	28	CPEO, EI, fatigue, GI problems	4978	nt.8469-nt.13447
3	M	39	16	CPEO, EI, fatigue, GI problems	4113	nt.11262-nt.15375
4	M	59	10	CPEO, EI, fatigue, ptosis	4978	nt.8469-nt.13447
5	M	45	29	CPEO, EI, fatigue, ptosis	7595	nt.7845-nt.15440
6	M	35	33	CPEO, EI, fatigue, GI problems	8039	nt.7637-nt.15676
7	M	31	16	CPEO, EI, fatigue, SVT	4978	nt.8469-nt.13447
8	F	34	9	CPEO, ptosis, mild dysphagia	5340	nt.6714-nt.12054

All patients displayed symptoms of myopathy, with CPEO and either ptosis or exercise intolerance (EI). All patients harboured a sporadically occurring single, large-scale mtDNA deletion, with precise mapping of the mtDNA deletion breakpoint showing that three patients (Patients 2, 4 and 7) had the 4977 bp common mtDNA deletion.

SVT, supraventricular tachycardia; GI, gastrointestinal tract.

Satellite cells were obtained from muscle biopsies with Fluorescently Activated Cell Sorting (FACS) using an antibody (CD56) which recognizes the adhesion molecule NCAM. Between 1251 and 36 136 CD56^+^ cells were isolated per biopsy, constituting 3–7% of the total cell number in the sample. In addition to collecting a homogenate sample of CD56^+^ cells, for Patient 7 it was possible to FACS sort single CD56^+^ cells into wells of a 96-well plate for further analysis. We had the opportunity to establish myoblast cultures from seven of the seventeen biopsies performed; in the other cases not enough tissue was available for cell extraction. When culturing primary myoblasts, fibroblast contamination is a common finding. To determine the myoblast purity of our cells, immunofluorescent detection of the myogenic marker desmin was performed. This revealed the percentage of myoblasts to be 84.5% ± 13.1 (SD) (Supplementary Material, Table S1).

### Quantification of single, large-scale mtDNA deletions in CD56^+^ cells

**Figure 1.**
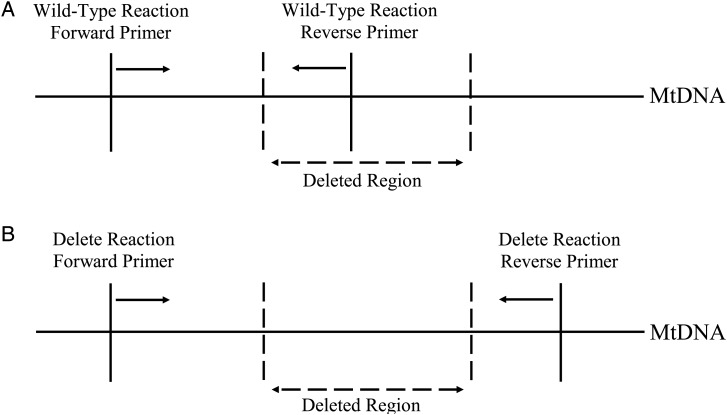
Deletion-specific SYBR^®^ Green qPCR. (**A**) Reaction designed to amplify only wild-type mtDNA, with primers chosen to anneal to regions within the breakpoints of the deletion. These primers will therefore not allow amplification of mtDNA containing a deletion. (**B**) The reaction designed to amplify mtDNA harbouring a deletion used primers that annealed to either side of the breakpoint. Owing to the extension time of the qPCR only mtDNA with a deletion would have an amplicon short enough to be amplified.

It was demonstrated using quantitative PCR (qPCR) methods, (MTND1/MTND4 TaqMan^®^ and SYBR^®^ Green) (Fig. 1), that all eight patients enrolled in the study harboured single mtDNA deletions in their CD56^+^ cells at levels very similar to those of mature muscle (Table 2). The average heteroplasmy level in CD56^+^ cells was 44.8 ± 23.5% (SD) (*n* = 17) and in the muscle homogenate it was 48.1 ± 17.5% (*n* = 8). A paired *t*-test revealed that there was no significant difference between the mtDNA deletion levels in CD56^+^ cells and in mature muscle homogenate samples (*P* = 0.48).

**Table 2.** MtDNA heteroplasmy levels in CD56^+^ cells and skeletal muscle (SKM) tissue

Patient	1		2			3			4		5			6	7		8
Biopsy	First	Second	First	Second	Third	First	Second	Third	First	Second	First	Second	Third	First	First	Second	Second
CD56^+^ Cells	46.6%	48.6%	54.8%	43.2%	60.8%	82.9%	79.2%	57.5%	49.6%	47.1%	0.6%	1.3%	3.6%	44.3%	50.1%	53.0%	39.7%
SKM	52.5%		58.4%			67.0%			44.7%		10.1%			48.4%	61.6%		42.8%

The MTND1/MTND4 TaqMan^®^ qPCR assay was used to quantify levels of single, large-scale mtDNA deletions in CD56^+^ cells and skeletal muscle samples of DNA with heteroplasmy levels >25%. Patient 5 was known to have a heteroplasmy level <25% and a SYBR^®^ Green qPCR assay specific to this patient's single mtDNA deletion was used (Supplementary Material, Table S2).

**Figure 2.**
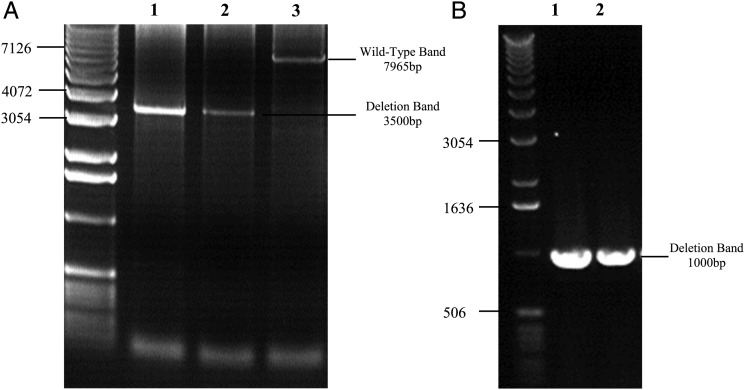
Long-range PCR gels demonstrating single mtDNA deletions in CD56^+^ cells after two rounds of amplification. (**A**) CD56^+^ cells from Patient 1 were amplified with primers (Supplementary Material, Table S3) to generate a 7965 bp mtDNA fragment. PCR products were then diluted 1:50 (lane 1) and 1:100 (lane 2) and amplified a second time with primers which would generate a 7063 bp fragment in a control DNA sample (lane 3). Bands of ∼3500 bp can be observed in the patient samples (lanes 1 and 2), confirming a deletion of ∼3500 bp which corresponds to that determined by breakpoint sequencing (Table 1). (**B**) Muscle homogenate (lane 1) and CD56^+^ cells (lane 2) from Patient 6 were amplified with primers to generate a 10 774 bp mtDNA amplimer. Products were diluted and re-amplified using PCR primers to generate a 8995 bp mtDNA fragment. Bands can be seen corresponding to an ∼1000 bp amplicon, which corresponds to the mtDNA deletion mapped in this patient that is 8039 bp (Table 1).

To confirm this finding, we investigated CD56^+^ cells from two patients (1 and 6) using long-range PCR. In both subjects, this showed a single, large-scale mtDNA deletion of appropriate size (Table 1), providing further confirmation that this mtDNA mutation was present in the satellite cells (Fig. 2).

### MtDNA deletions in myoblasts

Sufficient muscle tissue was available from five of the eight patients (Patients 2, 3, 4, 5 and 6) to establish primary myoblast cultures; from Patients 2 and 3 it was possible to establish myoblast cell cultures from two separate biopsies, giving a total of seven myoblast cultures that were investigated. Myoblasts were subcultured up to passage 6 or 7 before being differentiated into myotubes; however, this was not possible in all cases as some of the cells were growing extremely slowly. These cells were therefore differentiated at earlier passages. A small fraction of cells were retained after each passage to investigate the levels of patient-specific, single, large-scale mtDNA deletions (Table 3). Single, large-scale mtDNA deletions were not present in myoblasts from Patients 4, 5 and 6. However, myoblasts grown from two separate muscle biopsies from Patient 3 behaved very differently. The myoblasts grown from one biopsy contained no detectable levels of single, large-scale mtDNA deletion after the first passage, but myoblasts obtained from a subsequent biopsy harboured the mtDNA deletion. The myoblasts obtained from both muscle biopsies of Patient 2 contained a single, large-scale mtDNA deletion.

**Table 3.** MtDNA heteroplasmy levels in myoblasts and myotubes

Patient	2		3		4	5	6
Biopsy	First	Second	Second	Third	Second	First	First
Myoblasts Passage 1	ND	ND	0.75% ± 0.1	ND	0%	0%	ND
Myoblasts Passage 2	ND	ND	0%	61% ± 14	0%	0%	ND
Myoblasts Passage 3	ND	78.8% ± 0.9	0%	68% ± 9	0%	0%	0%
Myoblasts Passage 4	35% ± 1.6	79.5%1.0	0%	68% ± 7	ND	0%	0%
Myoblasts Passage 5	33% ± 1.0	81.25% ± 0.8	0%	56.9% ± 0.006	ND	0%	0%
Myoblasts Passage 6	34.75% ± 0.4	79% ± 1.4	ND	43% ± 12	ND	0%	0%
Myoblasts Passage 7	34% ± 4.0	ND	ND	23.5% ± 2.8	ND	0%	0%
Myotubes	22.25% ± 0.8	79.7% ± 0.004	0%	0%	0%	0%	0%

QPCR assays were used to quantify levels of mtDNA containing a deletion in myoblasts at different passages, and myotubes. Myoblasts from three of the seven cell cultures harboured single mtDNA deletions after the first passage, and when these cells were differentiated into myotubes, the deletion was lost completely from one of the cell cultures and reduced in another. It was not always possible to grow the cells up to passage 7 before differentiating them into myotubes due to slow growth; additionally some cell cultures were only obtained from the Biobanking facility after a number of passages.

Values are means ± SE.

ND, analysis not done.

Single, large-scale mtDNA deletions were therefore detected in three out of the seven myoblast cell cultures investigated. In two out of the three myoblast cell cultures that originally maintained the genetic defect, there was a gradual decline as the cells proliferated and differentiated into myotubes (Table 3). To determine whether the process of differentiation resulted in myogenic cells losing their mtDNA mutation, myoblasts from Patients 2 and 3 were differentiated into myotubes at an earlier passage (Table 4). However, this had no effect on their heteroplasmy level, suggesting it was the process of myoblast proliferation and not differentiation that caused the reduction in mtDNA deletion levels.

**Table 4.** The effect on mtDNA heteroplasmy levels of differentiating myoblasts into myotubes

Patient	Biopsy	Myoblasts Passage 4 (%)	Myoblasts Passage 5 (%)	Myotubes Passage 5 (%)	Myoblasts Passage 6 (%)
2	First	38	32	43	35
	Second	79	81	87	79
3	Third	75	57	51	31

Myoblasts from Patients 2 and 3, those that maintained mtDNA deletion levels in their replicating myoblasts, were differentiated into myotubes at passage 5. The mtDNA deletion levels in the myotubes were similar to those observed both before differentiation and in the myoblasts of a subsequent passage that did not undergo differentiation. This experiment was only performed on a single occasion.

In view of the persistence of a single, large-scale mtDNA deletion in myoblasts from two of our patients we wanted to exclude the possibility of an mtDNA duplication, which might explain the persistence of the mutation (26). A Southern blot, using *BamHI* and *SnaBI* (Supplementary Material, Fig. S1A) to digest mtDNA obtained from the myoblasts of Patient 2, showed no evidence of a duplication (Supplementary Material, Fig. S1B). In addition, since in patients with duplications the rearrangement often persists in replicating tissues (26), we checked for the presence of the single, large-scale mtDNA deletion in blood from Patients 2 and 3, and urine from Patient 2 and could not detect it (data not shown).

### Single cell investigations

**Figure 3.**
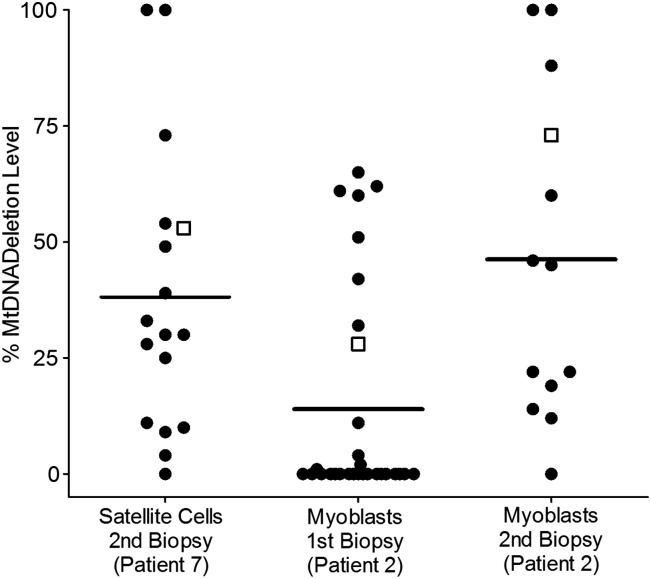
MtDNA heteroplasmy levels in single satellite cells (CD56^+^) and myoblasts. Single satellite cells were obtained from the second muscle biopsy of Patient 7 by FACS sorting, and myoblasts were obtained from two biopsies from Patient 2. Because of it's sensitivity to low quantities of DNA, samples were investigated using the common deletion SYBR^®^ Green qPCR (Supplementary Material, Table S2). Both satellite cells and myoblasts were heterogeneous based on mtDNA deletion levels. Lines indicate the mean heteroplasmy level of the single cells, while open squares show the heteroplasmy levels determined for the homogenate sample using the MTND1/MTND4 TaqMan^®^ qPCR.

It was decided to investigate whether the levels of single, large-scale mtDNA deletions were a result of all cells harbouring the same amount of mutation, or if satellite cells and myoblasts are a heterogeneous population based on single, large-scale mtDNA deletion levels. Single CD56^+^ cells from Patient 7 and single myoblasts from two biopsies of Patient 2 were FACS sorted into a 96-well plate. Both CD56^+^ cells and myoblasts were shown to be a heterogeneous population based on mtDNA deletion levels, with levels ranging from very high to very low (Fig. 3). FACS analysis can only be performed on live cells and, due to the timing of muscle biopsies, it was only possible to investigate single satellite cells and single myoblasts from these two patients.

### MtDNA copy number

**Figure 4.**
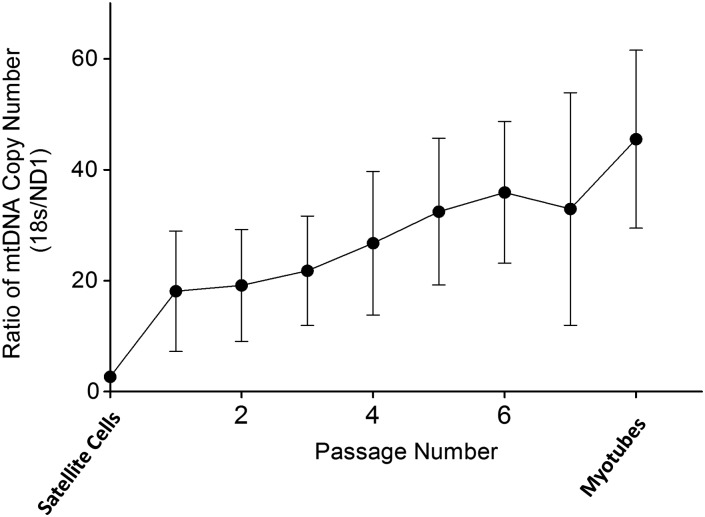
Relative mtDNA copy number in CD56^+^ cells and cultured myoblasts. Satellite cells contained low amounts of mtDNA. There was a gradual increase in the mtDNA copy number following activation, proliferation and conversion to myotubes. Values are means ± SE (*n* = 7).

The copy number of mtDNA was determined using qPCR to compare a nuclear marker, 18S rRNA, with the MTND1 region. In all the patients, the mtDNA copy number of CD56^+^ cells was low. While there was some fluctuation, there was an increase in the mtDNA copy number after satellite cell activation (Fig. 4), which increased further during myoblast proliferation.

## DISCUSSION

This research aimed to determine the reason for the loss of sporadically occurring mtDNA mutations from regenerating muscle cells in patients with mitochondrial myopathies (22–24). The main findings were that: (i) mtDNA mutations were present in satellite cells of all eight patients with myopathy caused by a sporadically occurring, single, large-scale mtDNA deletion at levels comparable with that observed in muscle; (ii) mtDNA deletions persisted in the myoblasts of only some patients; (iii) mtDNA deletions appear to be lost at two stages; firstly very early on during the initial myoblast proliferation phase or during satellite cell activation and secondly during later myoblast proliferation; (iv) muscle regeneration with incorporation of satellite cells remains a viable therapy in patients with sporadically occurring conditions, although the mechanisms around the process are somewhat different from that originally hypothesized. We believe this work not only has implications for the treatment of mitochondrial disease, but also adds to our knowledge regarding how mtDNA mutations segregate during development and are lost from certain cellular populations.

Satellite cells and skeletal muscle share a common embryological origin: they are both derived from the dermomyotome region of the somites that have budded off from the mesoderm during development (17). Our results demonstrating no significant difference in mtDNA heteroplasmy levels between muscle satellite cells and mature skeletal muscle are in some ways not surprising. While there is an eventual demarcation between muscle stem cells that form skeletal muscle and those that will form satellite cells, it would appear that the mutation has occurred and segregated to these cells before this separation. This would agree with previous studies showing that single, large-scale mtDNA deletions are present in the early embryo, as demonstrated by the same mtDNA deletion being identified in both members of a pair of identical twins (27). It is interesting to note that our study and others (14,15,22,24) have shown that sporadically occurring mtDNA mutations can be lost from replicating myoblasts during the process of muscle regeneration; however, the mutation has not been lost from replicating myoblasts during embryological development. At this stage, we can only speculate on this; it is possible that the mature muscle is a more hostile environment than embryologically developing muscle and therefore the selection pressures are stronger, or maybe during development muscle precursor cells do not divide as many times as required to select against the mutation.

When individual satellite cells from Patient 7 were investigated, they showed variable levels of mutated mtDNA, ranging from very high to very low. This is interesting because it suggests that there is very little, if any, selection for or against single, large-scale mtDNA deletions in the satellite cells. Satellite cells are largely quiescent and contain very few mitochondria. The extreme variation in mutation load suggests either an mtDNA genetic bottleneck during embryogenesis during the formation of satellite cells, or random drift of mutation load with time.

Given the previous findings of little or no mtDNA mutation in the myoblasts of patients with sporadically occurring mitochondrial diseases, it was expected that the mtDNA mutation would be absent from the cultured myoblasts of our patients. However, we observed remarkable differences not only between patients, but in one case between biopsies from the same patient, in the heteroplasmy levels of cultured myoblasts. In the majority of cultures, the mtDNA mutation was rapidly lost. In two of the three cases where the single, large-scale mtDNA deletion was detected in the myoblasts, the mutation levels decreased as the cells headed towards differentiation. Single myoblasts from both of the biopsies from Patient 2 were heteroplasmic for single, large-scale mtDNA deletions. This has been reported in a previous investigation that demonstrated the heteroplasmic status of clonal myoblasts from patients with single, large-scale mtDNA deletions and Kearns–Sayre syndrome (16).

The differences in the behaviour of the myoblasts from Patient 2 are intriguing, with one cell culture showing increased levels of mtDNA mutation when compared with mature muscle and satellite cells, and one with lower levels. The age and heteroplasmy levels in this patient were no different from other patients, and the deletion was the so-called ‘common deletion’ (4), which was also present in Patients 4 and 7 in our study. Why this patient behaves so differently is unknown but highlights that segregation of mtDNA mutations between cells is not straightforward and likely to represent both the selection pressures and nuclear genome effects. Interestingly, this patient had no evidence of the single, large-scale mtDNA deletion in blood or urine (data not shown) and thus selection had occurred in other tissues.

When culturing myoblast primary cells, fibroblast contamination is a common occurrence, and while we demonstrated a reasonably high level of myoblast purity, we still had some fibroblast contamination. Fibroblasts have previously been shown to be free of mtDNA mutations in patients with sporadically occurring mitochondrial diseases (14,15); this may have resulted in a lower observed mtDNA deletion load in the myoblasts of our patients. While satellite cells were originally defined according to their anatomical location (28), it is now clear they are a heterogeneous population based on their expression of different markers. Most previous work in satellite cell biology has been performed on mice, and there is therefore a lack of specific markers and thus commercially available antibodies for human satellite cells (21). This study used CD56 (NCAM) to isolate satellite cells because it is a marker that identifies myogenic cells in humans; in addition, there is a good, commercially available, directly conjugated antibody for this marker. Reports have demonstrated the presence of NCAM in various components of the blood (29), but these cells are unlikely to contaminate in view of the washing prior to FACS sorting and also the fact that in blood samples sporadically occurring, single, large-scale mtDNA deletions are often absent.

It would appear that regenerating muscle cells are subjected to two periods of selection, with the first phase occurring very early on before myoblasts were assessed for mutation levels. It is difficult to determine how selection occurred at this stage as it is possible that the mutations might have been lost at the point of satellite cell activation or that the mutation might have been lost from the myoblasts themselves during the early stages of proliferation before we were able to assess mutation levels. Our data suggest a very small amount of mtDNA is present in satellite cells and thus during replication into myoblasts selection pressure may be extreme. If this were the case, it would explain why the single, large-scale mtDNA deletion is lost so early in the majority of patient's myoblasts. Alternatively or complementary to this, an asymmetric division of the satellite cell after activation (30) could result in one cell acquiring more or less of the disease-causing mutation. Depending on which cell proceeds to form myoblasts and which returns to repopulate the stem cell pool, the mutation may be maintained or lost from the regenerating muscle cells. The second selection point was a more gradual selection against myoblasts with high levels of mtDNA deletion, whereby cells with high levels of mutation were unable to proliferate as well.

An approach currently being investigated to help patients suffering from mitochondrial myopathy caused by sporadically occurring mtDNA mutations is resistance training. The purpose of resistance training is 2-fold, to increase muscle strength and to incorporate cells harbouring lower or no mutated mtDNA (31). The ‘proof of principle’ of gene shifting has already been established (22–24). This current research suggests that the mechanism of action may be different from that originally hypothesized. Rather than the sporadically occurring mutation being absent from satellite cells, it is present, but subsequently lost over two selection points. This would suggest that resistance exercise training still remains a viable therapy for the majority of patients with sporadically occurring mtDNA mutations. However, it is important to note that selection pressures in a cell culture environment differ from the mature muscle *in vivo* and thus the behaviour of proliferative muscle cells may vary. While one myoblast culture appeared to have entered into a steady-state regarding mutation levels, the two cultures that decreased single mtDNA deletion levels required a number of passages for this to occur. Our primary cell cultures were propagated for around seven passages. While our two cell cultures that reduced in mtDNA mutation load did so over these seven passages, it is possible that *in vivo* they may not have as many divisions in which selection can occur. This highlights the need for further research in these patient groups along the lines of that previously performed (14,22,24,25) and with primary human myoblast cultures to better characterize the regenerative response to satellite cell activation, and reveal if there are any patient characteristics that determine whether patients maintain, lose or even increase the mutation in their regenerating muscle cells.

In conclusion, we have shown that mtDNA mutations are present in the muscle satellite cells of patients with mitochondrial myopathies due to sporadically occurring, single, large-scale mtDNA deletions. In the majority of the patients, the deletion is lost during the transition from satellite cell to myoblast. For those mutations found in the myoblasts, there is often gradual loss of the mtDNA deletion with subsequent passages. Thus, activation of satellite cells remains a viable option for patients with sporadically occurring conditions; however, the mechanisms behind the process are different from that originally hypothesized.

## MATERIALS AND METHODS

### Subjects and samples

Ethical approval for this study was obtained from the Newcastle and North Tyneside Local Research Ethics Committees and all investigations were performed after informed consent was obtained.

Muscle biopsies (*vastus lateralis*) were obtained from eight patients [two females aged 46 ± 17 (SD) years; six males aged 42.5 ± 9.9 years] harbouring a sporadically occurring, single, large-scale mtDNA deletion. All the patients presented with CPEO and the majority had exercise intolerance and fatigue (Table 1).

### Isolation of satellite cells

Muscle samples were stored in solution A (30 mm HEPES, 130 mm NaCl, 3 mm KCL, 11 mm Glucose, Phenol Red), until the isolation of satellite cells could be performed. Muscle samples were dissociated using a Medimachine (BD Biosciences) according to the manufacturer's instructions. Approximately 25 mg of muscle and 500 µl of phosphate buffered saline was placed in a Medicon (BD Biosciences) and put into the Medimachine for 20 s. The blade inside the Medicon dissociated the sample and allowed the cells to pass through a sieve into a collection chamber, thus separating them from the connective tissue. The cells were filtered through a 30 µm filter (CellTrics^®^, Partec) and then counted using a Beckman Coulter Cell Viability Analyser.

Phycoerythin (PE) labelled Mouse Anti-Human CD56 IgG_1_ (BD Pharmingen™) (NCAM) was incubated with the sample (10 µl per million cells) for 30 min in the dark. The sample was then washed using a BD Lyse Wash Assistant (BD Biosciences).

CD56 positive (CD56^+^) cells were isolated from the sample using FACS on a FACSAria machine (BD Biosciences). The sample was excited at 488 nm and the fluorescence registered at 585 nm. The sample was collected in either a microcentrifuge tube or in 96-well plates and then centrifuged at 166*g* for 5 min. The supernatant was discarded and the cells stored at −20°C until required.

### Cell culture

Myoblast cell cultures were established by the MRC Centre for Neuromuscular Diseases Biobank Newcastle, according to established protocols (32). Muscle samples were stored in solution A and cultures established within 3 days. Myoblasts were cultured in Skeletal Muscle Growth Medium (PromoCell) supplemented with 10% foetal calf serum, 1.5% l-glutamine and 300 µl of gentamicin. Differentiation of myoblasts to myotubes was performed by changing to a low serum medium (98% DMEM and 2% horse serum) for 7 days. Differentiation was determined to be successful if cells were visible containing two or more nuclei. Harvesting and passaging of cells was carried out by detaching the cells with trypsin and centrifuging at 166*g* for 5 min; the supernatant was discarded, leaving a cell pellet which was either frozen at −20°C or re-plated.

### Desmin labelling

To determine myoblast purity, cells were grown on cover slips and labelled with monoclonal mouse anti-human desmin (DAKO). Alexa Fluor^®^ −488 goat anti-mouse secondary antibody was applied and the cells mounted with VECTASHIELD^®^ Mounting Medium with DAPI. Counts were performed on an Axiovert 200M (Zeis), using AxioVision Release 4.6.3 software (Zeiss) with myoblast purity being determined by the number of desmin^+^ DAPI^+^ cells compared with the number of Desmin^−^ DAPI^+^ cells.

### DNA extraction

DNA was extracted from homogenate samples of CD56^+^cells, myoblasts and myotubes using a Qiagen DNeasy Blood and Tissue Kit according to the manufacturer's instructions. DNA was eluted in 40 µl of dH_2_O and stored at −20°C until required. Single cell DNA extraction was performed using lysis medium consisting of 50 µl of 500 nm Tris–HCL, 250 µl of 1% Tween 20, 190 µl of dH_2_O and 5 µl of proteinase K (Invitrogen), of which 15 µl was added to each cell. The cells were then incubated at 55°C for 2 h and 95°C for 10 min, and stored at 4°C until required (9).

### MtDNA investigations

#### Taqman^®^ QPCR assay

To detect and quantify single, large-scale mtDNA deletions, the MTND1/MTND4 qPCR multiplex TaqMan^®^ assay was used (33,34). This method uses primers and fluorescent probes to bind to the *MTND1* (Forward nt.3485-nt.3504, Reverse nt.3532-nt.3553, Probe nt.3506-nt.3529) and *MTND4* (Forward nt. 12087-nt.12109, Reverse nt.12140-nt.12170, Probe nt.12111-nt.12138) regions of the mtDNA. All of the patients in this study had the *MTND4* region at least partly removed by their mtDNA deletion, while the *MTND1* sequence was spared in all cases. The proportion of mtDNA harbouring a deletion was determined by the established ΔCT method (34). Samples were run in triplicate on an ABI Step One Plus Real Time PCR System (Applied Biosystems), along with control samples of mtDNA with known heteroplasmy levels.

Relative mtDNA copy number was determined by comparing the nuclear marker 18 s rRNA to the preserved *MTND1* region in a TaqMan^®^ assay. Primers and probes were used to amplify the *MTND1* (as above) and the 18s rRNA sequence (Forward 5′-GCC GCT AGA GGT GAA ATT CTT G-3′, Reverse 5′-CAT TCT TGG CAA ATG CTT TCG-3′, probe 5′-CCG GCG CAA GAC GGA CCA GA-3′). Reactions were performed in a 25 µl volume, consisting of 12.5 μl of TaqMan^®^ Universal MasterMix, 0.75 μl of *MTND1* forward and reverse primer (10 µm), 0.5 μl of probe (5 µm), and 7.5 μl of dH_2_O and 1 µl of sample DNA. The 18s rRNA master mix was identical to *MTND1* except with 0.13 μl of forward primer (10 μm) and 10.12 μl of dH_2_O. The two reactions were set up in parallel, with each patient's CD56^+^ cells, myoblasts and myotubes being run together; blood and muscle DNA were included as controls. The reaction conditions were 2 min at 50°C, 10 min at 95°C, 40 cycles of 15 s at 95°C and 60 s at 60°C. The MtDNA copy number was calculated using the equation R = 2^−ΔCt^ (34).

#### SYBR^®^ green QPCR assays

Using primers specific to mtDNA breakpoint sequences, it is possible to design quantitative real-time PCR assays specific for an individual's single, large-scale mtDNA deletion. These specific assays have been able to accurately quantify very low mtDNA deletion levels (27), using very small amounts of DNA. This assay was therefore used to assess the level of mtDNA heteroplasmy in a patient with low levels of mtDNA deletion (Patient 5) and to confirm the absence or to quantify low levels in the satellite cells and myoblasts of other patients. Patient 5 had previously been shown to harbour low levels of a single mtDNA deletion (9%) in his muscle based on Southern blotting (Rare Mitochondrial Disorders of Adults and Children Diagnostic Service, Newcastle upon Tyne). This is below the sensitivity level of the MTND1/MTND4 TaqMan^®^ qPCR assay (34). In addition, Patients 3, 4 and 6 were initially shown, using the MTND1/MTND4 TaqMan^®^ qPCR to have at least one myoblast cell culture with mtDNA deletion levels below the sensitivity of this assay. A further two SYBR^®^ Green assays were therefore developed (Supplementary Material, Table S2) and one assay re-optimized (27) to determine the exact amount of delete mtDNA, if any, in these cells.

Primers were designed using the Primer3 software (v.0.4.0) (35) to amplify either wild-type or mutant (deleted) mtDNA. The wild-type primers were designed to only amplify wild-type mtDNA as the reverse primer binding site was within the mtDNA breakpoints of the deletion (Fig. 1A). Owing to the qPCR reaction conditions the delete mtDNA primers would only amplify DNA fragments if an mtDNA deletion of several Kb was present (Fig. 1B). A standard curve for each assay was constructed using wild-type mtDNA and delete mtDNA from the patient with a known heteroplasmy level. The ΔCT for delete and wild-type mtDNA was calculated for the samples and compared with the standard curve to determine the exact heteroplasmy level.

Reactions were performed on an ABI Step One Plus Real Time PCR System (Applied Biosystems) using Platinum^®^SYBR^®^Green qPCR SuperMix-UDG (Invitrogen) in a 25 µl volume, with 1 µl of sample DNA. The cycling conditions were 95°C for 10 min, followed by 40 cycles of 95°C for 15 s, and then the relevant annealing temperature for 1 min (Supplementary Material, Table S2); this was followed by a melt curve analysis to confirm the specificity of the amplified product. Fluorescence levels were acquired at the end of each cycle.

### Long-range PCR

Long-range PCR was performed on CD56^+^ cells from Patients 1 and 6 using two rounds of amplification (36). The first round of amplification for Patient 1 used primers (Supplementary Material, Table S3) to generate a 7965 bp mtDNA fragment and for Patient 6 the primers generated a 10 774 bp product. Products were diluted in dH_2_O and amplified a second time. Patient 1 had primers designed to give a final 7063 bp product and Patient 6 had primers to give a final fragment of 8995 bp. Amplified products were separated on 0.7% agarose gels.

### Southern blot

Rearrangements of mtDNA were screened for mtDNA duplications in total DNA extracted from myoblasts of Patient 2 using a Southern blot, employing the enzymes *BamHI* and *SnaBI,* with the *SnaBI* cutting within the deletion breakpoints and enabling detection of any duplicated molecules*.* Products were probed with a PCR-generated probe that hybridized against the non-coding control region (37).

### Statistical analysis

Data were analysed using GraphPad Prism and Microsoft Excel. Anderson-Darling tests were used to test for normality and means were compared with two sample *t*-tests.

## SUPPLEMENTARY MATERIAL

Supplementary Material is available at *HMG* online.

*Conflict of Interest statement*. None declared.

## FUNDING

This work was supported by the Medical Research Centre for Neuromuscular Diseases, Newcastle University Centre for Brain Ageing and Vitality supported by the Biotechnology and Biological Sciences Research Council, Engineering and Physical Sciences Research Council and the Medical Research Council as part of the cross-council Lifelong Health and Wellbeing Initiative (G0700718), The Wellcome Trust Centre for Mitochondrial Research (906919) and Newcastle NIHR Biomedical Research Centre. Funding to pay the Open Access publication charges for this article was provided by RCUK.

## Supplementary Material

Supplementary Data

## References

[1] Larsson N.G., Oldfors A. (2001). Mitochondrial myopathies. Acta Physiol. Scand..

[2] Holt I.J., Harding A.E., Morgan-Hughes J.A. (1988). Deletions of muscle mitochondrial DNA in patients with mitochondrial myopathies. Nature.

[3] Taylor R.W., Turnbull D.M. (2005). Mitochondrial DNA mutations in human disease. Nat. Rev..

[4] Moraes C.T., DiMauro S., Zeviani M., Lombes A., Shanske S., Miranda A.F., Nakase H., Bonilla E., Wernegk L.C., Servidei S. (1989). Mitochondrial DNA deletions in progressive external ophthalmoplegia and Kearns-Sayre Syndrome. N. Engl. J. Med..

[5] Rossignol R., Malgat M., Mazat J.P., Letellier T. (1999). Threshold effect and tissue specificity. Implication for mitochondrial cytopathies. J. Biol. Chem..

[6] Rossignol R., Faustin B., Rocher C., Malgat M., Mazat J.-P., Letellier T. (2003). Mitochondrial threshold effects. Biochem. J..

[7] Taylor R.W., Schaefer A.M., Barron M.J., McFarland R., Turnbull D.M. (2004). The diagnosis of mitochondrial muscle disease. Neuromuscul. Disord..

[8] Old S.L., Johnson M.A. (1989). Methods of microphotometric assay of succinate dehydrogenase and cytochrome c oxidase activities for use on human skeletal muscle. Histochem. J..

[9] Murphy J.L., Ratnaike T.E., Shang E., Falkous G., Blakely E.L., Alston C.L., Taivassalo T., Haller R.G., Taylor R.W., Turnbull D.M. (2012). Cytochrome c oxidase-intermediate fibres: importance in understanding the pathogenesis and treatment of mitochondrial myopathy. Neuromuscul. Disord..

[10] Blackwood J.K., Whittaker R.G., Blakely E.L., Alston C.L., Turnbull D.M., Taylor R.W. (2010). The investigation and diagnosis of pathogenic mitochondrial DNA mutations in human urothelial cells. Biochem. Biophys. Res. Commun..

[11] Aure K., Ogier de Baulny H., Laforet P., Jardel C., Eymard B., Lombes A. (2007). Chronic progressive ophthalmoplegia with large-scale mtDNA rearrangement: can we predict progression?. Brain.

[12] McShane M.A., Hammans S.R., Sweeney M., Holt I.J., Beattie T.J., Brett E.M., Harding A.E. (1991). Pearson syndrome and mitochondrial encephalomyopathy in a patient with a deletion of mtDNA. Am. J. Hum. Genet..

[13] Holt I.J., Harding A.E., Cooper J.M., Schapira A.H., Toscano A., Clark J.B., Morgan-Hughes J.A. (1989). Mitochondrial myopathies: clinical and biochemical features of 30 patients with major deletions of muscle mitochondrial DNA. Ann. Neurol..

[14] Fu K., Hartlen R., Johns T., Genge A., Karpati G., Soubridge E.A. (1996). A novel heteroplasmic tRNA^leu(CUN)^ point mutation in a sporadic patient with mitochondrial encephalomyography segregates rapidly in skeletal muscle and suggests an approach to therapy. Hum. Mol. Genet..

[15] Weber K., Wilson J.N., Taylor L., Brierley E., Johnson M.A., Turnbull D.M., Bindoff L.A. (1997). A New mtDNA mutation showing accumulation with time and restriction to skeletal muscle. Am. J. Hum. Genet..

[16] Moraes C.T., Schon E.A., Dimauro S., Miranda A.F. (1989). Heteroplasmy of mitochondrial genomes in clonal cultures from patients with Kearns–Sayre syndrome. Biochem. Biophys. Res. Commun..

[17] Gros J., Manceau M., Thome V., Marcelle C. (2005). A common somitic origin for embryonic muscle progenitors and satellite cells. Nature.

[18] Le Grand F., Rudnicki M.A. (2007). Skeletal muscle satellite cells and adult myogenesis. Curr. Opin. Cell Biol..

[19] Hawke T.J., Garry D.J. (2001). Myogenic satellite cells: physiology to molecular biology. J. Appl. Physiol..

[20] Kuang S., Kuroda K., Le Grand F., Rudnicki M.A. (2007). Asymmetric self-renewal and commitment of satellite stem cells in muscle. Cell.

[21] Boldrin L., Mutoni F., Morgan J.E. (2010). Are human and mouse satellite cells really the same?. J. Histochem. Cytochem..

[22] Clark K.M., Bindoff L.A., Lightowlers R.N., Andrews R.M., Griffiths P.G., Johnson M.A., Brierley E.J., Turnbull D.M. (1997). Reversal of a mitochondrial DNA defect in human skeletal muscle. Nat. Genet..

[23] Shoubridge E.A., Johns T., Karpati G. (1997). Complete restoration of a wild-type mtDNA genotype in regenerating muscle fibres in a patient with a tRNA point mutation and mitochondrial encephalomyopathy. Hum. Mol. Genet..

[24] Taivassalo T., Fu K., Johns T., Arnold D., Karpati G., Soubridge E.A. (1999). Gene shifting:a novel therapy for mitochondrial myopathy. Hum. Mol. Genet..

[25] Murphy J.L., Blakeley E.L., Schaefer A.M., He L., Wyrick P., Haller R.G., Taylor R.W., Turnbull D.M., Taivassalo T. (2008). Resistance training in patients with single, large-scale deletions of mitochondrial DNA. Brain.

[26] Poulton J., Deadman M.E., Bindoff L., Morten K., Land J., Brown G. (1993). Families of mtDNA re-arrangements can be detected in patients with mtDNA deletions: duplications may be a transient intermediate form. Hum. Mol. Genet..

[27] Blakely E.L., He L., Taylor R.W., Chinnery P.F., Lightowlers R.N., Schaefer A.M., Turnbull D.M. (2004). Mitochondrial DNA deletion in ‘identical’ twin brothers. J. Med. Genet..

[28] Mauro A. (1961). Satellite cell of skeletal muscle fibres. J. Cell Biol..

[29] Lanier L.L., Le A.M., Civin C.I., Loken M.R., Phillips J.H. (1986). The relationship of CD16 (Leu-11) and Leu-19 (NKH-1) antigen expression on human peripheral blood NK cells and cytotoxic T lymphocytes. J. Immunol..

[30] Kuang S., Gillespie M.A., Rudnicki M.A. (2008). Niche regulation of muscle satellite cell self-renewal and differentiation. Cell Stem Cell.

[31] Taivassalo T., Haller R.G. (2004). Implications of exercise training in mtDNA defects—use it or lose it?. Biochem. Biophys. Acta.

[32] EuroBioBank (2004). http://www.eurobiobank.org/en/services/services.htm.

[33] Krishnan K.J., Bender A., Taylor R.W., Turnbull D.M. (2007). A multiplex real-time PCR method to detect and quantify mitochondrial DNA deletions in individual cells. Anal. Biochem..

[34] He L., Chinnery P.F., Durham S.E., Blakeley E.L., Wardell T.M., Borthwick G.M., Taylor R.W., Turnbull D.M. (2002). Detection and quantification of mitochondrial DNA deletions in individual cells by real-time PCR. Nucleic Acids Res..

[35] Rozen S., Skaletsky H. (2000). Primer3 on the WWW for general users and for biologist programmers. Methods Mol. Biol..

[36] Reeve A.K., Krishnan K.J., Elson J.L., Morris C.M., Bender A., Lightowlers R.N., Turnbull D.M. (2008). Nature of mitochondrial DNA deletions in substantia nigra neurons. Am. J. Hum. Genet..

[37] Rotig A., Bourgeron T., Chretien D., Rustin P., Munnich A. (1995). Spectrum of mitochondrial DNA rearrangements in the Pearson marrow-pancreas syndrome. Hum. Mol. Genet..

