# Supplementary material for: Mitochondrial DNA deletions in muscle satellite cells: implications for therapies
Source: Hum Mol Genet. 2013 Jul 11;22(23):4739–47. doi: 10.1093/hmg/ddt327 (PMC3820134; doi:10.1093/hmg/ddt327)
Supplement: Supplementary Data [file supp_22_23_4739__index.html]

Mitochondrial DNA deletions in muscle satellite cells: implications for therapies — Mitochondrial DNA deletions in muscle satellite cells: implications for therapies — Supplementary Data 

# Mitochondrial DNA deletions in muscle satellite cells: implications for therapies

## 

Supplementary Data

**Files in this Data Supplement:**

- Supplementary Data - Doc file
