# Supplementary material for: Mitochondrial DNA deletions in muscle satellite cells: implications for therapies
Source: Hum Mol Genet. 2013 Jul 11;22(23):4739–47. doi: 10.1093/hmg/ddt327 (PMC3820134; doi:10.1093/hmg/ddt327)

| **Patient** | **Biopsy** | **Myoblast Percentage** |
| --- | --- | --- |
| **3** | **2^nd^** | **98%** |
|  | **3^rd^** | **76%** |
| **4** | **2^nd^** | **98%** |
| **5** | **1^st^** | **67%** |
| **6** | **1^st^** | **82%** |

**Supplementary Table 1** The percentage of cells within myoblast cultures that stained positive for desmin. Myoblasts were grown on cover slips and stained for desmin, a myogenic marker. The number of cells staining positive for both DAPI (nuclei) and desmin are given as a percentage of all cells with a DAPI positive nuclei.

|  | **Wild Type Reaction Primers** | | | **Delete Reaction Primers** | | |  |
| --- | --- | --- | --- | --- | --- | --- | --- |
| **Patient** | **Nucleotide Position** | **Concentration** | **MgCl_2_** | **Nucleotide Position** | **Concentration** | **MgCl_2_** | **Annealing Temp** |
| 3 | nt.11155-nt.11173 (F)  nt.11287-nt.11267 (R) | 125pmo (F) 125pmol (R) | 3mM | nt.11155-nt.11173 (F)  nt.15384-nt.15366 (R) | 125pmo (F) 125pmol (R) | 4mM | 64^o^C |
| 4 | nt. 8430-nt.8449 (F)  nt.8524-nt.8505 (R) | 125pmo (F) 125pmol (R) | 3mM | nt.8419-nt.8438 (F)  nt.13511-nt.13493 (R) | 125pmo (F) 125pmol (R) | 4mM | 52.3^o^C |
| 5 | nt.7816-nt.7834 (F)  nt.7881-nt.7863 (R) | 75pmol (F), 100pmol (R) | 4mM | nt.7816-nt.7843 (F),  nt.1544-nt.15463 (R) | 100pmol(F) 75pmol (R) | 3mM | 56^o^C |
| 6 | nt.7592-nt76109 (F),  nt.7680-nt7660 (R) | 125pmo (F) 125pmol (R) | 3mM | nt.7592-nt.7610 (F)  nt.15717-nt15735 (R) | 125pmo (F) 100pmol (R) | 3mM | WT 56^o^C  Delete 55^o^C |

**Supplementary Table 2** Reaction conditions for deletion specific mtDNA SYBR Green Real Time assays. Patient 4 harboured the common deletion and this assay was therefore also used for samples of myoblasts from patient 2 and 7. (F) and (R) refer to forward and reverse primers respectively. Reactions to quantify wild-type and mtDNA harbouring a deletion were set up in parallel and optimised individually for primer concentrations, MgCl_2_ concentration and annealing temperature.

| **Long Range PCR Primers** | | | |
| --- | --- | --- | --- |
| **Patient** |  | **Forward Primers (5’-3’) Nucleotide Number** | **Reverse Primers (5’-3’) Nucleotide Number** |
| 1 | First Amplification | 6863-6882  ATTTAGCTGACTCGCCACAC | 14857-14838  AAGGAGTGAGCCGAAGTTTC |
|  | Second Amplification | 7272-7293  GGCTCATTCATTTCTCTAACAG | 14374-14356  AGGATTGGTGCTGTGGGTG |
| 6 | First Amplification | 5855-5875  AGATTTACAGTCCAATGCTTC | 129-110  AGATACTGCGACATAGGGTG |
|  | Second Amplification | 6863-6882  ATTTAGCTGACTCGCCACAC | 15896-15877  TACAAGGACAGGCCCATTTG |

**Supplementary Table 3** Primers used to amplify mtDNA from CD56^+^ cells. Two rounds of long range PCR amplification were performed on samples, with the primers used in the second round sitting within those of the first.

**Supplementary Figure 1** Southern Blot on cells from patient 2. MtDNA obtained from myoblasts from patient 2 and a control subject was digested using restriction endonucleases that cut at nt.14257 (*BamH1*) and nt.10735 (*SnaBI*). A. In the mtDNA from the patient the *BamHI* enzyme which cuts outside the mtDNA deletion shows a small amount of wild-type mtDNA, and a stronger single band of DNA harbouring a deletion that runs lower than the 16.5kb band. *SnaB1* which cuts within the deletion does not identify any additional bands compared to the BamH1 and therefore no duplicated species. B. In the control sample cut with *BamH1* a single WT band of 16.5Kb can be observed, in the same mtDNA cut with *SnaBI* again a wild-type linear band can be observed, however this enzyme has not have fully digested the mtDNA as there is still a super-coiled fragment visible.


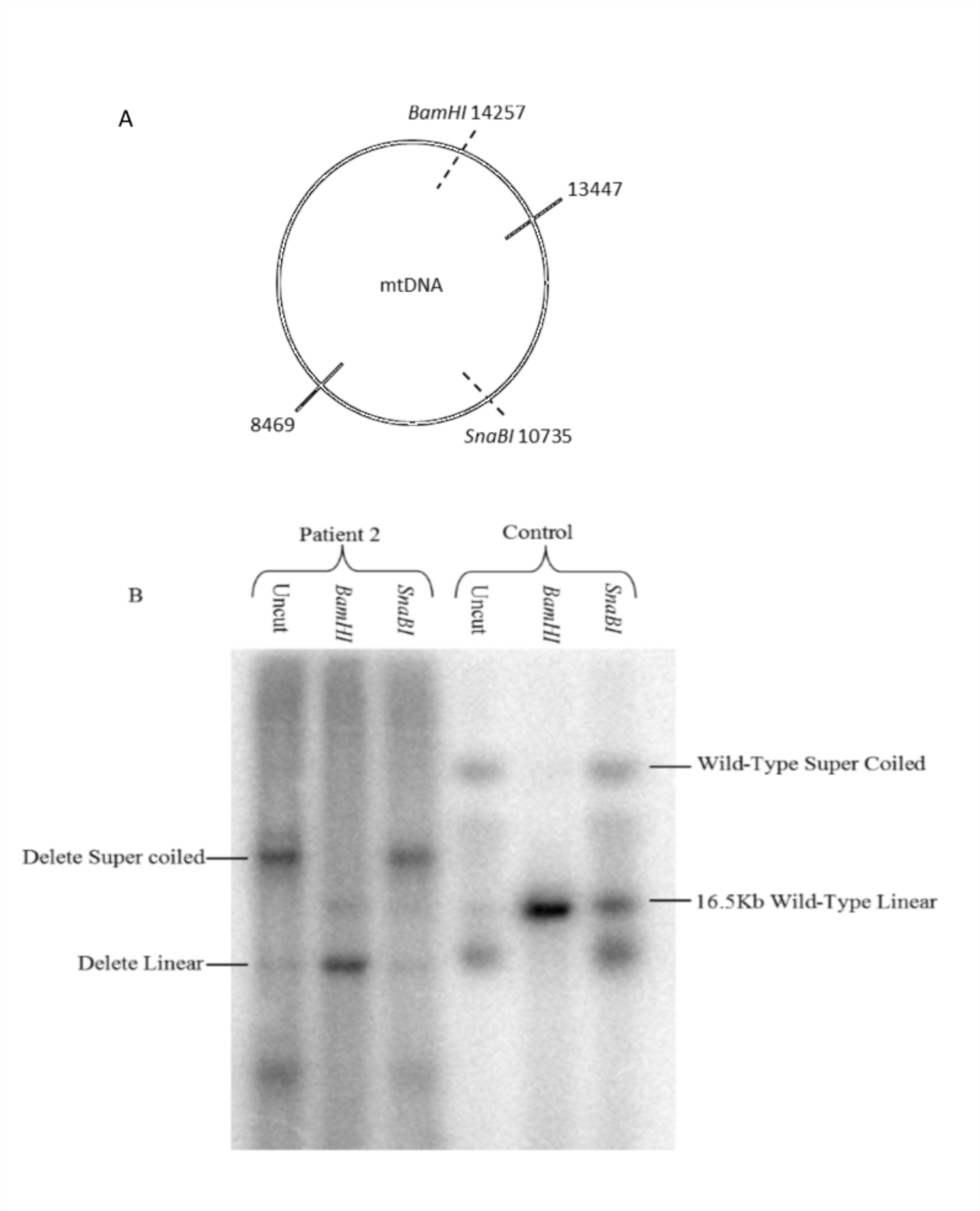

Supplement: Supplementary Data [file supp_ddt327_ddt327supp.doc]
